# Metastatic Renal Cell Carcinoma to the Soft Tissue 27 Years after Radical Nephrectomy: A Case Report

**DOI:** 10.3390/medicina59010150

**Published:** 2023-01-12

**Authors:** Caterina Marra, Luigi Losco, Alessandra Ceccaroni, Paola Pentangelo, Donato Troisi, Carmine Alfano

**Affiliations:** 1 Plastic Surgery Unit, Department of Medicine, Surgery and Dentistry, University of Salerno, Baronissi, 84081 Salerno, Italy; 2 ORL Unit, Azienda Ospedaliera Universitaria OO.RR. San Giovanni di Dio e Ruggi d’Aragona, Via S. Leonardo 1, 84131 Salerno, Italy

**Keywords:** metastatic, renal cell carcinoma, clear cell renal cell carcinoma, head and neck cancer, subcutaneous tissue, metastasis, soft tissue

## Abstract

*Background and Objectives*: Approximately 20–40% of patients affected with renal cell carcinoma (RCC) develop either distant metastatic or locally recurring disease following radical nephrectomy. Soft tissue, skin, and the central nervous system are less common metastatic sites. We present the case of a patient who has received a diagnosis of RCC; it was found that she had no metastases at the time of nephrectomy but had metastases in the soft tissue and subcutaneous tissue of the scalp 27 years later. As far as we can tell, this is the longest period elapsed between primary renal tumor and subcutaneous/soft tissue metastasis; moreover, this case is the first report of a combined soft tissue/subcutaneous metastasis from RCC. *Case presentation*: A 73-year-old woman underwent right radical nephrectomy 27 years earlier for clear cell renal cell carcinoma (CCRCC). She presented at our unit because she noticed swelling in the left temporal region; after radiological exams, a benign lesion was suspected. The patient underwent surgical eradication, but the massive bleeding did not allow the removal of the lesion. A biopsy of the mass was performed and the histological examination was consistent with RCC metastases. *Conclusions*: Metastases from renal cell carcinoma to the subcutaneous and soft tissues are rare. It is essential to take into account RCC metastases in the differential diagnostic of the new starting mass of the head and neck, and the necessity for close and continuous surveillance of patients diagnosed with renal cancer even after a long disease-free period should be emphasized.

## 1. Introduction

Renal cell carcinoma (RCC) is the most common type of renal tumor and the most lethal among urological tumors. It currently accounts for 90% of all renal tumors and approximately 2–3% of adult malignancies [1,2]. Clear cell renal cell carcinoma (CCRCC) is the most frequent histological type. RCC has an aggressive clinical course due to a strong tendency to metastasize [3]. Approximately 20–40% of patients develop remote metastasis or a locally recurrent disease after a radical nephrectomy; the metastatic disease is usually reported within 5 years of the oncologic surgery [4,5]. Metastases are more common in males, with the most frequent metastases pulmonary parenchyma (50–60%), bone (30–40%), liver (30–40%), and brain (5%). Soft tissue, skin, and the central nervous system are less common metastatic sites [6,7,8,9].

We present the case of a patient who has received a diagnosis of RCC; it was found that she had no metastases at the time of nephrectomy but had metastases in the soft tissue and subcutaneous tissue of the scalp 27 years later.

## 2. Case Presentation

**Figure 1:**
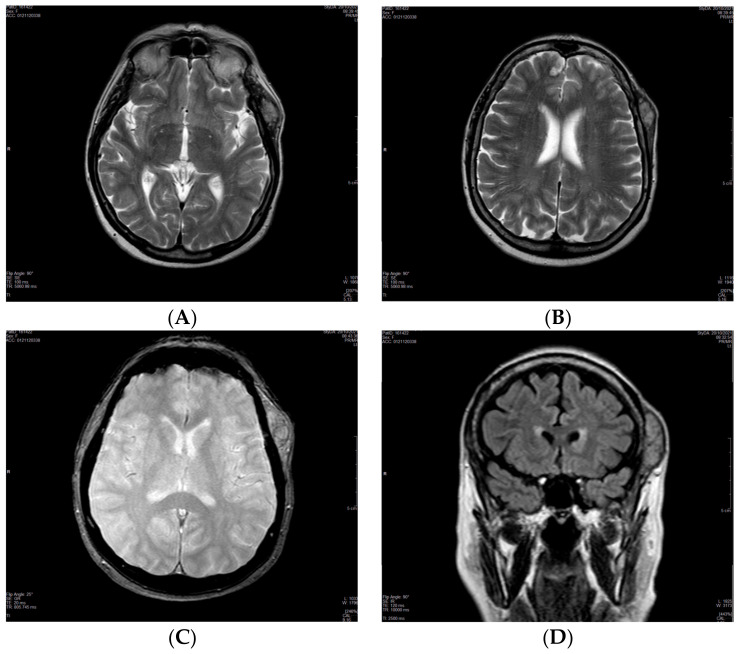
MRI in the axial (**A**–**C**) and coronal plane brain (**D**) shows a single 2.5 × 5 cm soft tissue temporal lesion in the left side with moderate contrast impregnation (**C**).

**Figure 2:**
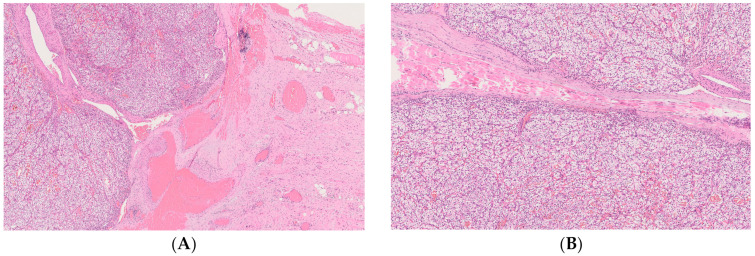

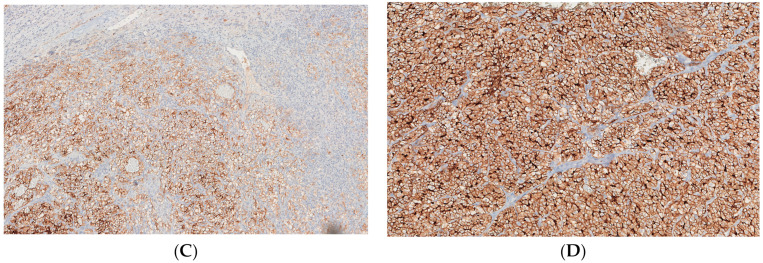
Histopathological examination showing a metastatic lesion consistent with RCC. (**A**,**B**) Hematoxylin and eosin staining; (**C**,**D**) CD10+.

A 73-year-old female was introduced to our unit with a history of CCRCC; she underwent right radical nephrectomy 27 years earlier, with the initial tumor stage pT2G2Nx. The surveillance was performed as follows: blood tests, chest CT, and abdomen-pelvis MRI were performed every 3 months for the first 2 years and then every 6 months up to 5 years post-nephrectomy. After the 5-year follow-up, imaging and blood tests were performed every year. Follow-up regularity was only impaired during the COVID-19 pandemic outbreak. In November 2020, the patient complained of a swelling in the left temporal region for approximately 2 months without other symptoms. An ultrasound examination was requested by the general practitioner: A hypoechoic oval mass lesion measuring 30 × 10 mm with intense vascular signal to the Power Doppler was revealed. Afterwards, she underwent an otolaryngological examination: the presence of a rounded neoformation with a soft consistency, smooth and uniform surface, and movable on the deep planes was reported, and it was diagnosed as a possible sebaceous cyst. Surgical excision was recommended, but the patient refused due to both the ongoing COVID-19 pandemic and personal reasons. A year later, she came to our department because she noticed that the subcutaneous mass had enlarged. This past year, no treatment or other clinical examinations were performed. At this point, the mass appeared hard and not movable on the deep planes. Surgical treatment was indicated as a result of the late development of the lesion and its fast growth. Magnetic resonance imaging (MRI) of the brain with and without contrast media was required. A single 2.5 × 5 cm soft tissue temporal lesion, with moderate contrast impregnation, was shown in the temporal left side: a benign desmoid-type lesion was suspected by the radiologist (Figure 1). In November 2021, the patient underwent surgery to remove the lesion. The massive bleeding and the texture of the lesion did not allow the removal. A biopsy was performed, but the pathologist did not provide a diagnosis due to the small sample. A thin needle aspiration of the lesion guided by ultrasound was then requested: a hemorrhagic background with a few clusters of cells with large vacuolized cytoplasm and nuclear atypia was showed on cytological examination. Subsequently, in February 2022, a surgical biopsy of the lesion was performed. Immunohistochemical tests on tumor cells respond positively to CD10. A histological piece was stained with hematoxylin and eosin; the examination revealed the presence of fibroadipose and muscle tissue with diffused infiltration from clear cell renal cell carcinoma. RCC metastasis was reported due to specific cytological and histological features (Figure 2). The patient was referred to the Cancer Board; a total body CT documented the absence of involvement of other sites, including the remaining left kidney, so it was possible to exclude a de novo disease with metastases. The case was reviewed and pharmacological treatment started. Pazopanib 200 mg, a protein kinase inhibitor, was offered to the patient. At first, four pills of 200 mg each per day were prescribed. However, following the occurrence of blood pressure peaks, the patient had to reduce the dosage to one pill of 200 mg per day and two pills of 200 mg per day, on alternate days. In addition, the patient had to monitor her blood pressure daily in a blood pressure diary. This therapy is still ongoing, and a clinical exam of the palpable mass revealed a reduction in consistency and size up to 2 × 4.3 cm; however, a radiological follow-up has not been performed yet (Table 1).

## 3. Discussion

Most malignant tumor metastases are reported within 5 years of the original diagnosis of the primary tumor. Nevertheless, “late metastases” do happen. According to scientific reports, “late” is generally defined as >10 years after the primary tumor has been removed and without evidence of a local recurrence at the primary tumor site. Such inactivity has been explained through the entrance of tumor cells in a dormant state. Cell dormancy may be defined as a non-productive state or an interruption in the cell cycle leading to an extended G0 phase [10]. However, dormancy, unlike senescence, is a reversible state, and several reasons have been hypothesized to cause the end of a dormant state and subsequent metastasization; among others, immune system impairment, dietary ingredients, and psychological distress may play a key role [11,12,13]. Nephrectomy is the treatment of choice for RCC without any metastasis. Nephrectomy offers a 5-year survival rate of more than 90% in patients with stage I RCC and approximately 40% in patients with stage II and III RCC [14]. RCC rarely metastasize to the cutaneous, subcutaneous, and soft tissue, and different mechanisms are outlined for visceral tumor metastases. The most common is direct invasion of the cutaneous tissue overlying the malignant mass; moreover, the rich vascular structure of RCCs encourages hematogenous extension and the development of remote metastases. The most relevant blood-borne extension pathway for RCC is the cava vein system: tumoral embolization via the Batson plexus, specifically the anastomosis between the avalvular spine and the epidural venous system, seems to be the preferred path [15]. It is believed that arteriovenous and systemic shunts facilitate the movement of tumor cells towards the head and neck [16]. The most commonly reported primary carcinomas causing clinically recognized soft tissue metastases are those of the lung, kidney, and colon [17]. Regarding the cutaneous metastasis, several studies reported that approximately 3% of renal tumors metastasized to the skin, and the most frequent location was the scalp, followed by the abdominal area [18]. They typically consist of simple lesions of rapid growth, ranging in diameter from a few millimeters to a few centimeters, appearing as clearly delineated subcutaneous nodules or infiltrating plaques, which may be painful or painless. They may also be described as a pulsatile mass and vary from fleshy to purplish-colored [5]. According to Motzer et al., the survival of RCC metastatic patients ranges from 10.2 months to 22 months [19]. However, the development of skin metastasis in RCC is associated with poor prognosis, and the majority of patients die less than 6 months after metastasis is detected [20]. The vast majority of patients with a metastatic soft tissue mass died on average less than 9 months and no later than 3 years post-diagnosis, although additional patients were reported to be alive with disease at longer follow-up durations of up to 5 years [16]. As a consequence, treatment options are limited and palliative for the most part [20].

In the present case, the interval of nephrectomy for RCC and emergence of soft tissue metastasis was as long as 27 years. This time period is long; indeed, metastasis after nephrectomy typically occurs within 3 to 5 years [21,22]. However, Kim et al. [22] reported that after an initial 5-year postoperative disease-free interval, 15% of patients developed distant metastases during the ensuing 10 years, suggesting that long-term surveillance should continue, though the optimal surveillance regimen is yet to be defined. In their series, primary tumor size, stage, and histologic subtype were associated with late recurrence. In our report, the stage and CCRCC subtype suggested a higher risk of recurrence according to Kim et al. Subcutaneous metastases are very uncommon and, as far as we know, the longest time elapsed between primary renal tumor and subcutaneous metastasis is 15 years [23]. For all we know, this is the first combined reporting case of soft tissue and subcutaneous metastasis from RCC [3,7,15,17,18,24,25]. The infiltration from CCRCC involved muscular and subcutaneous tissue. Patients who have a history of RCC require lifelong monitoring and follow-up, and any soft tissue lesion should not be neglected. RCC can present with atypical sites of metastasis; soft tissue metastases, as in our case, without involvement of the skin are more subtle and difficult to diagnose, especially in the early stages if the mass is mobile. However, a timely diagnosis could make surgical removal easier and improve the patient’s prognosis. It is pivotal to take into account RCC soft tissue metastasis in the differential diagnosis of new starting nodules, ulcers, lipomas, cysts, or tumors with or without a vascular aspect in the head and neck; in fact, this region is a common localization for primary tumors, and the diagnosis could be misleading [26,27,28,29].

The treatment of metastatic renal cancer is always controversial, as large series of metastasectomies are reported in the literature, but little is known about the management of metastasis in atypical sites [24]. Metastatic renal cell carcinoma (mRCC) is usually unresponsive to chemotherapy and is only moderately susceptible to radiation therapy. Since it is one of the most immunogenic tumors, immunotherapy with interferon alpha (IFNa) or interleukin-2 (IL-2) is often used as first-line therapy [25]. A combination of immunotherapy (IL-2) with radiotherapy (RT) has demonstrated synergy in preclinical studies [30,31]. However, if metastases are present, targeted treatment is often the first option. The target agents routinely administered are tyrosine kinase inhibitor, vascular endothelial growth factor inhibitor, or mTOR inhibitor [32]. In our case, the patient is receiving ongoing biological therapy, and the effectiveness of the treatment is under evaluation. Moreover, periodic tests are performed to evaluate the toxicity of the treatments.

## 4. Conclusions

An uncommon case of metastatic kidney carcinoma was presented; the soft tissue and subcutaneous metastasis was reported 27 years after nephrectomy. A patient with a diagnosis of RCC should undergo proper monitoring even after a long disease-free survival. Extended postoperative surveillance protocols for RCC after nephrectomy should be advocated.

## Figures and Tables

**Table 1:** Timeline of patient’s medical history.

Time	Event
1993	Right radical nephrectomy caused by renal cell carcinoma.
November 2020	Swelling in the left temporal region for 2 months.
24 November 2020	Ultrasonography: hypoechoic oval mass lesion measuring 30 × 10 mm.
27 November 2020	Otolaryngological examination: soft and movable mass lesion was reported. Surgical excision was refused by the patient.
October 2021	Plastic surgery visit due to the growth of the mass: hard and non-movable mass.
20 October 2021	MRI of the brain: single 2.5 × 5 cm soft tissue temporal lesion. Suspected benign lesion.
2 November 2021	Surgical resection attempted and not accomplished due to massive bleeding.
13 January 2022	Cytological examination without diagnosis.
February 2022	Biopsy of the lesion: metastasis of renal cell carcinoma.
7 March 2022	The patient was directed to the Cancer Board for evaluation.
5 April 2022	Total body CT scan: absence of involvement of other sites.
June 2022	Drug treatment with Pazopanib 800 mg/daily started. Dose was reduced to 200 mg/daily and 400 mg/daily on alternate days due to blood pressure peaks.

## Data Availability

No new data were created or analyzed in this study. Data sharing is not applicable to this article.
